# Trimethylamine N-oxide (TMAO) in human health

**DOI:** 10.17179/excli2020-3239

**Published:** 2021-02-11

**Authors:** Paulina Gatarek, Joanna Kaluzna-Czaplinska

**Affiliations:** 1 Institute of General and Ecological Chemistry, Faculty of Chemistry, Lodz University of Technology, Lodz, Poland

**Keywords:** trimethylamine N-oxide, TMAO, trimethylamine, TMA, analytical technique, human health, diseases

## Abstract

Due to numerous links between trimethylamine-N-oxide (TMAO) and various disorders and diseases, this topic is very popular and is often taken up by researchers. TMAO is a low molecular weight compound that belongs to the class of amine oxides. It is formed by the process of oxidation of trimethylamine (TMA) by the hepatic flavin monooxygenases (FMO1 and FMO3). TMAO is mainly formed from nutritional substrates from the metabolism of phosphatidylcholine/choline, carnitine, betaine, dimethylglycine, and ergothioneine by intestinal microflora in the colon. Its level is determined by many factors, such as age, gender, diet, intestinal microflora composition, kidney function, and also liver flavin monooxygenase activity. Many studies report a positive relationship between the level of TMAO concentration and the development of various diseases, such as cardiovascular diseases and cardiorenal disorders, including atherosclerosis, hypertension, ischemic stroke, atrial fibrillation, heart failure, acute myocardial infarction, and chronic kidney disease, and also diabetes mellitus, metabolic syndrome, cancers (stomach, colon), as well as neurological disorders. In this review, we have summarized the current knowledge on the effects of TMAO on human health, the relationship between TMAO and intestinal microbiota, the role of TMAO in different diseases, and current analytical techniques used in TMAO determination in body fluids.

## Introduction

Trimethylamine N-oxide (TMAO) is a dietary component that belongs to the class of amine oxides with the formula (CH_3_)_3_NO. TMAO is an oxidized form of trimethylamine (TMA) (Gessner et al., 2020[22]; Subramaniam and Fletcher, 2018[67]; Ufnal et al., 2015[74]). One of the functions of TMAO is the ability to affect the structure and activity of a large group of biologically important compounds. TMAO acts as an important stabilizer of the protein folded state and nucleic acid. Thermodynamic studies on the effects of TMAO on proteins have shown that TMAO prevents protein denaturate and counteracts the effects of pressure and heat (Ufnal et al., 2015[74]). According to the literature, there are several mechanisms that can explain the folding propensity of TMAO, however, they are still not fully understood (Mondal et al., 2013[49]; Sarma and Paul, 2013[62]). 

The aim of this review was to summarize the current state of knowledge about TMAO, biological properties, metabolic pathways, and essential metabolism nutrients which act as precursors of TMAO. The importance of TMAO metabolism and its role in human health are also highlighted. We provided and discussed the potential relationships between TMAO and intestinal microbiota. Attention was also paid to the potential contribution of intestinal microbiota-derived production of TMAO from the metabolism of nutrients, which has been associated with an increased risk of major adverse disorders in humans. Then we presented the potential role of TMAO in the etiology of various diseases, and the possible mechanisms that could explain their association. Finally, we analyzed the current analytical techniques which are used in TMAO determination in biological fluids.

## TMA/TMAO Metabolism in the Human Body

TMA is formed from the dietary compounds present in the diet, which is then immediately absorbed and converted into TMA by various enzymes (Wang et al., 2019[79]; Zeisel and Warrier, 2017[89]). It is mainly formed from nutritional substrates from the metabolism of phosphatidylcholine/choline, carnitine, betaine, dimethylglycine, and ergothioneine by intestinal microflora in the colon. TMA is absorbed into the bloodstream and transformed into TMAO by hepatic flavin monooxygenases (FMO1 and FMO3), but it also can be degraded to methylamine, dimethylamine (DMA), and ammonia within the colon (Velasquez et al., 2016[76]; Subramaniam and Fletcher, 2018[67]; Gessner et al., 2020[22]; Papandreou et al., 2020[56]). A diet rich in such nutritional substrates has a higher choline and carnitine content, which are precursors to the formation of TMA and TMAO (Ufnal et al., 2015[74]). The highest amounts of TMAO from food products is in the saltwater fish containing about 3 g/kg of this compound (Mitchell et al., 2002[45]). Moreover, it is indicated that the formation of TMA and TMAO accompanying the consumption of eggs is dose-dependent, there is a threshold concentration of choline which must be ingested in order to be converted into TMAO. In addition to the precursors mentioned above, carnitine related metabolites, such as γ-butyrobetaine and crotonobetaine, also betaine, which is a choline oxidation product, is one of the important precursors. Some of these compounds may participate in the formation of TMA and TMAO depending on intestinal microbiota and can be also generated by L-carnitine metabolism (Wang et al., 2019[79]).

**Figure 1:**
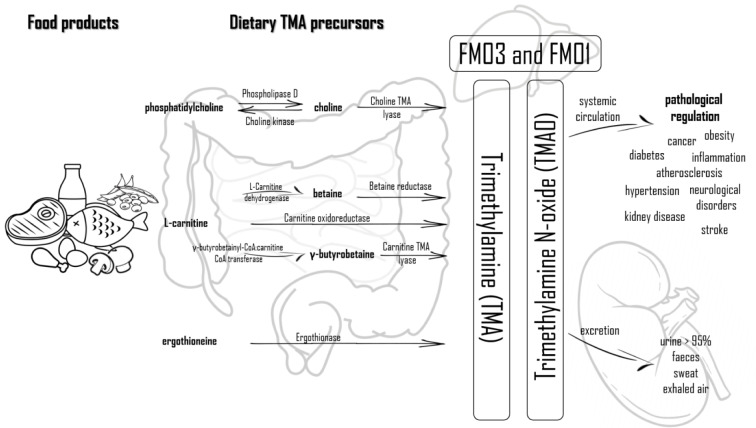
Biochemical pathways leading to the production and metabolism of TMA and TMAO and the resulting health consequences

Food products rich in phosphatidylcholine, which is considered to be the main dietary source of choline and consequently TMAO, are eggs, liver, milk, meat (red meat, poultry), and fish (Wang et al., 2011[80]; Subramaniam and Fletcher, 2018[67]). A major precursor for TMA generation in vegetarians, vegans, and omnivores alike is phosphatidylcholine, which is the main dietary source of choline. Red meat is rich in carnitine, while beef and other meats (poultry), liver, fish, and egg yolks are rich in choline. Soya, vegetables (cauliflower and cabbage) and whole grains are other food sources of choline. Furthermore, in high concentrations, choline is present in dietary supplements and drug (products improving heart and liver function, anti-dementia drugs) in the form of phosphatidiocholine. Phosphatidiocholine can be converted into choline by the Phospholipase D enzyme, and inversely, choline transformed into phosphatidiocholine catalyzed by choline kinase. Choline is transformed into TMA by the choline TMA lyase enzyme. Found mainly in plants, betaine is reduced to TMA by betaine reductase in a coupled reduction-oxidation reaction (Janeiro et al., 2018[28]). The two enzymes (choline dehydrogenase and betaine aldehyde dehydrogenase), enable the conversion of choline into betaine (Fennema et al., 2016[19]). One of the main precursors of TMA is also L-carnitine. The conversion of L-carnitine to TMA is done by carnitine oxidoreductase or can be converted into two other precursors like betaine (by L-carnitine dehydrogenase) and γ-butyrobetaine (GBB) (by the γ-butyrobetainyl-CoA:carnitine CoA transferase enzyme) (Fennema et al., 2016[19]; Janeiro et al., 2018[28]). Ergothioneine can be another source of TMA obtained from dietary sources, such as some types of beans, meat products (liver and kidney) or mushrooms. Ergothioneine is converted into TMA by the ergothionase enzyme (Janeiro et al., 2018[28]). Figure 1(Fig. 1) presents biochemical pathways leading to the production and metabolism of TMA and TMAO, and the resulting health consequences.

After absorption, most of TMA (nearly 95 %) is oxidized to TMAO, which is transported to the tissues for accumulation as an osmolyte or, more frequently, cleared by kidneys (Velasquez et al., 2016[76]), which is then excreted, mainly with urine in a 3:95 TMA:TMAO ratio within 24h. Other ways of excretion of TMAO are excretion with sweat, faeces (4 %), exhaled air (less than 1 %) or other body secretions (Papandreou et al., 2020[56]). Methanogenic bacteria containing the TMAO demethylase enzyme can metabolize TMAO to DMA, formaldehyde, ammonia and methane (Chhibber-Goel et al., 2016[10]). The exact mechanism of TMAO excretion from the human body is shown in Figure 1(Fig. 1). Moreover, it has been shown that TMAO from food products can be directly absorbed in the gut (Zhang et al., 1999[90]; Cho et al., 2017[11]). Therefore, the levels of plasma TMAO are influenced by the formation of TMA and its degradation as well as the secretion rate of TMA, DMA, and TMAO (Gessner et al., 2020[22]).

## The Relationship between TMAO and Intestinal Microbiota

The intestinal microbiota is shaped from birth and plays a key role in the immune system. One of the main functions of the intestinal microbiota is to form systemic immunity and maintain immune homeostasis. Factors affecting the intestinal microbiota are diet, type of labor, antibiotic use during infancy, and age, because the composition of the intestinal microbiota changes with age.

Interest in the study of the concentration of bacterial metabolites, including TMAO, in mental disorders and other diseases has significantly increased in recent years. Although TMAO has been known for a long time, for the first time in 2011, Wang et al. suggested that TMAO could be harmful to human health (Wang et al., 2011[80]). An increase in TMAO concentration may be caused by diet, changes in the composition of intestinal microflora, gut dysbiosis, or impairment of the gut-blood barrier. To increase our knowledge of the composition of the intestinal microbiota and their contribution to the health status and disease, the development of sequencing technology has been beneficial. One of the key factors which influences the composition of the intestinal microbiota is diet. From various food sources the intestinal microbiota produces metabolites, such as short-chain fatty acids (SCFAs) and TMAO, which are associated with an increased risk of cardiovascular disease and mortality. The group of SCFAs includes acetate, propionate, and butyrate, which have blood pressure lowering properties, cardiac hypertrophy and fibrosis. In the last decade alone, at least 1000 gut bacterial species were reported, of which *Firmicutes, Bacteroidetes, Actinobacteria, Proteobacteria, Fusobacteria *and *Verrucomicrobia* are the dominant phyla, with *Firmicutes* and *Bacteroidetes* accounting for over 90 % of the gut bacteria (Muralitharan and Marques, 2020[51]). Bacteria species like *Clostridia, Proteus, Shigella* and *Aerobacter* are involved in the production of TMA (Subramaniam and Fletcher, 2018[67]).

Some research suggests that TMAO may cause proinflammatory responses and renal toxicity. The higher ratio of *Firmicutes* to *Bacteroidetes* demonstrating a greater response to the dietary precursor of TMAO which suggests that the production of TMAO may be a function of individual differences in the intestinal microbiota (Subramaniam and Fletcher, 2018[67]). Studies on mice have shown that intestinal bacteria are essential to convert dietary compounds to TMA (Zeisel and Warrier, 2017[89]). The production of TMA and TMAO can be almost completely suppressed using broad spectrum antibiotics, and after one month of withdrawal of antibiotics, the TMAO concentration returns to normal (Janeiro et al., 2018[28]).

Understanding the role of human intestinal microbiota has led to the identification of a large number of metabolites which are produced in the gut. These metabolites may play a role in human health and possible diseases. One of these is TMAO, whose elevated concentration increases the risk of developing renal failure, diabetes mellitus, heart failure, atherosclerosis, hypertension, metabolic syndrome, dyslipidemia which may lead to serious cardiovascular events (Al-Rubaye et al., 2019[2]).

The literature suggests that the enzymatic activity of TMA producing intestinal bacteria may promote the development of atherosclerosis due to the fact that TMA is easily absorbed from the intestinal tract and concerted in the liver into TMAO. Research conducted by Wang et al. (2011[80]) suggested that the level of TMAO is strongly associated with atherosclerosis (Wang et al., 2011[80]). 

In the gut some bacteria species like *Acinetobacter* can convert lecithin and carnitine into TMAO. Analysis of fecal specimens suggested that participants with enriched bacteria of the genus *Prevotella*, as a result of a high-fat diet, were characterized by higher levels of TMAO compared to participants with the enrichment of the genus *Bacteroides* (Yin et al., 2015[87]).

The intestinal microflora is also significantly affected by dysbiosis in a harmful way. By reducing or increasing the amount of TMA-producing strains within the microbiome, dysbiosis may alter TMAO levels (Yin et al., 2015[87]). As it is common knowledge, dysbiosis is triggered by unhealthy diet mainly high-animal fat diet. Moreover, dysbiosis contributes to the progression of CVDs by promoting atherosclerosis and hypertension. Kidney disease may be also caused by dysbiosis, due to the increased permeability intestinal barrier for metabolites produced by the intestines (Miller, 2013[46]). Griffin et al. (2019[23]) suggested that the increased concentration of TMAO may be correlated with a dysbiotic microbiome and inversely correlation between abundance of *Akkermansia mucinophilia* in colon biopsies and concentration levels of TMAO (Griffin et al., 2019[23]).

Numerous studies indicate that intestinal microbiota is involved in the pathogenesis and progression of various cardiovascular diseases such as heart failure (HF). HF causes changes in the composition of the intestinal microflora, which may affect the circulating levels of TMAO in human body. Researchers suggested intestinal strains, such as *Firmicutes* and *Proteobacteria*, which are capable of producing TMA. The strains of these bacteria show an increased proportion in patients with HF. This indicates that changes in intestinal microbiota may affect TMAO levels by regulating TMA synthesis in the intestines (Zhang et al., 2021[91]).

## Effects of Gender and Diet on TMAO Level

As we know, many factors influence the level of TMAO concentration in the human body. Such factors include gender, diet, gut microbiome composition, and kidney function. Unfortunately, there is no clear answer if gender can have an influence on TMAO concentrations. Some studies indicate a relationship between gender and metabolite concentration in a healthy study group (Obeid et al., 2017[53]; Manor et al., 2018[42]; Barrea et al., 2019[6]), while others do not (Wang et al., 2014[81]; Rohrmann et al., 2016[61]). Another very important factor influencing the levels of TMAO in the body is diet. A diet rich in products containing large amounts of precursors of TMA is associated with higher levels of TMAO in human body. The process of TMA formation from dietary products depends on the presence of gut microbes which are capable of metabolizing TMA precursors (Cho et al., 2017[11]; Manor et al., 2018[42]; Roberts et al., 2018[60]; Gessner et al., 2020[22]). Excessive consumption of food containing phosphatidylcholine and choline should be avoided, because these compounds cause increased production of TMAO. Due to the lower amount of L-carnitine and choline consumed along with food, in vegetarian and vegan population reduced levels of TMAO were observed (Koeth et al., 2013[32]). Intestinal microbiota produced higher levels of TMAO in omnivore participants, due to the increased consumption of L-carnitine mainly from red meat (Koeth et al., 2013[32]). Also high urinary TMAO excretion was observed in response to meat intake (Stella et al., 2006[64]), as well as the increased consumption of food high in choline such as eggs. The consumption of 2 or more eggs per day is associated with high concentration TMAO in plasma and urine (Miller et al., 2014[45]). In another study, the consumption of 3 eggs per day for 4 weeks resulted in lower density lipoprotein cholesterol (LDL-c)/HDL-c ratio, increasing high-density lipoprotein cholesterol (HDL-c), and elevated plasma concentrations of choline, without changing the concentration of TMAO in plasma (Bergeron et al., 2016[8]).

Additionally, a diet rich in fibers and vegetarian diet may result in the reduction of total choline intake. A diet considered to be healthy which contains significant amounts of saltwater fish and seafood leads to increased concentrations of plasma TMAO (Tang et al., 2013[72]). Higher levels of TMAO in plasma were associated with a low-carbohydrate diet and high resistant starch content. 

Griffin et al. (2019[23]) examined if the Mediterranean diet could reduce TMAO concentrations. They measured levels of TMAO before and after dietary intervention in 115 healthy subjects with increased risk of colon cancer. The diet was based on an increased intake of fiber and a change in the intake of many other dietary products containing fat to increase the intake of monounsaturated fats in the diet. They observed no significant changes in the levels of TMAO in plasma or in the ratio of precursor compounds of TMAO, but the Mediterranean diet may counteract the pro-inflammatory effects of increased TMAO generation (Griffin et al. 2019[23]).

In another study, Van Hecke and colleagues (2016[75]) examined the effect of red vs. white meat consumption on oxidative stress, inflammation and TMA concentration in rats. The diet rich in red meat resulted in higher concentration of urinary TMA and TMAO compared to the diet rich in white meat (chicken) (Van Hecke et al., 2016[75]).

Moreover, the high-salt diet is indicated as a cardiovascular risk factor. High salt intake increases the level of TMAO concentration in plasma, which is associated with the reduction of urinary TMAO excretion (Bielinska et al., 2018[9]). Moreover, excessive salt intake has an impact on the composition of intestinal bacteria, which suggests that the consumption of salt has a direct impact on the interaction between intestinal bacteria and their host homeostasis (Bielinska et al., 2018[9]).

Some studies indicate that fish intake would contribute to the increased production of TMAO. In the research conducted by Lenz et al. (2004[35]) the profiles of urinary metabolomics Swedish and British population were compared. They showed that the Swedish population was characterized by a higher urinary excretion of TMAO due to the consumption of fish-based foods, which was not observed in the British population that avoided fish intake 24 hours before the study (Lenz et al., 2004[35]). Similar results obtained by Dumas et al. (2006[16]) indicated that regular consumption of fish in the Japanese population resulted in increased levels of TMAO in urine (Dumas et al., 2006[16]). Lloyd et al. (2011[40]) observed an association between salmon intake and urinary TMAO excretion (Lloyd et al., 2011[40]). The diet rich in fish affects the concentration of TMAO, also the high-fat and high-calorie diet increases the levels of serum TMAO (Li et al., 2012[36]). All these data suggest that the composition of the diet is one of the most important factors increasing TMAO levels in the human body.

## Is there a Link between TMAO and Modern Diseases?

One of the metabolites produced by the intestinal microflora is TMAO. Researchers pay a lot of attention to the intestinal microbiome because of its possible role as a promoter of chronic diseases, cancers and even neurological disorders (Janeiro et al., 2018[28]). Intestinal microflora is connected with new age disorders like obesity (Musso et al., 2010[52]), insulin resistance (Musso et al., 2010[52]; Diamant et al., 2011[15]; Miele et al., 2015[44]; Tai et al., 2015[69]), atherosclerosis (Dalla Via et al., 2020[13]), cardiovascular diseases (ischemic stroke) (He et al., 2020[26]; Schneider et al., 2020[63]), as well as type 2 diabetes (Tai et al., 2015[69]), kidney failure (Bain et al., 2006[4]; Tang et al., 2015[70]; Missailidis et al., 2016[47]; Stubbs et al., 2016[66]; Mafune et al., 2016[41]), neurological disorders (Cryan and Dinan, 2012[12]), and cancer (stomach, colon) (Erdman and Poutahidis, 2015[18]; Dey and Ciorba, 2016[14]; Wang et al., 2019[79]). TMAO was also associated with mortality and hospitalization for cardiorenal disorders, including atrial fibrillation (Tang et al., 2014[71]), heart failure (Trøseid et al., 2015[73]), acute myocardial infarction (Suzuki et al., 2017[68]), and chronic kidney disease (Tang et al., 2015[70]).

Until now, high concentrations of TMAO have been combined with the development of atherosclerosis, which is one of the major causes of CVD. Increased concentrations of TMAO and also TMAO precursor in plasma have been observed in participants at risk of CVD (Janeiro et al., 2018[28]). The literature also suggests a correlation between high concentrations of plasma TMAO and the risk of developing atherosclerosis (Stubbs et al., 2016[66], Dalla Via et al., 2020[13]). On the other hand, however, recent studies indicate that TMA, rather than TMAO, affects the etiology of cardiovascular disorders. An increased cardiovascular risk in subjects with elevated plasma concentrations of TMAO depends on the increased level of plasma TMA (Jaworska et al., 2019[29]). TMAO affects also cholesterol metabolism in the bile acid compartments (Wang et al., 2011[80]). New research suggests that TMAO affects lipid and hormonal homeostasis and thereby possibly contributes to the development of CVD. Macrophage influx of cholesterol is activated by high concentration of blood TMAO. This process leads to foam cell formation and ultimately atherosclerotic lesions (Bennett et al., 2013[7]). TMAO shows an important role in cholesterol metabolism and metabolic stress under cholesterol overload. Intracellular cholesterol is stored with endoplasmic reticulum (ER). Disruption of cholesterol homeostasis influences the functioning of ER, which is leading to ER stress. To protect against ER stress, unfolded protein response is activated. The apoptosis process will be triggered when adaptive reactions fail to compensate. The results obtained by Zhao and colleagues (2019[92]) indicate that ER stress was decreased by TMAO intervention, possibly due to the reduction of cholesterol by TMAO treatment. TMAO may also mediated in the alleviation of ER stress (Zhao et al., 2019[92]). 

Research conducted by Wilson et al. (2015[83], 2016[82]) indicated lower levels of plasma TMAO in patients with inflammatory bowel disease (IBD) (2.27 μM) compared with the healthy control group. Lower levels of TMAO were also observed in subjects with ulcerative colitis (UC) (1.56 μM) compared to inactive disease (3.40 μM) (Wilson et al., 2015[83], 2016[82]). 

In research conducted by He et al. (2020[26]) 451 people aged 65 or older took part. The aim of this study was to identify the relationship between the levels of plasma TMAO and frailty in older people with cardiovascular disease (CVD) using UPLC-MS/MS. They found that in frail participants the levels of TMAO were significantly higher compared to non-frail participants, 4.04 µM vs. 3.21 µM, respectively. Researchers suggested that elevated levels of TMAO are independently associated with frailty among older adults with CVD (He et al., 2020[26]). As it is well known the levels of TMAO correlate with the risk of CVD, but some conflicting data suggested a specific role of this compound in ischemic stroke. 

Schneider et al. (2020[63]) analyzed the time course (on admission, after 48 h, after 3 months) of the levels of plasma TMAO in stroke patients compared with controls. Significantly higher levels of TMAO on admission were in stroke patients (n = 196, 4.09 µmol/L) compared to the control group (n = 100, 3.16 µM). After 48 h in stroke participants TMAO levels decreased significantly, but increased again after 3 months, while no changes were observed in the control group. The study indicated the importance of the time course of TMAO levels after ischemic stroke (Schneider et al., 2020[63]). A similar study was conducted by Rexidamu et al. (2019[59]). They investigated the levels of serum TMAO in 255 patients with ischemic stroke and 255 healthy controls. Higher levels of TMAO in stroke patients (5.8 μM) then in healthy volunteers (3.9 μM) were determined. Additionally, the increased risk of stroke was associated with an increase in TMAO concentration by 1 μM. Increased risk of first ischemic stroke and worse neurological deficit in participants were associated with higher levels of TMAO (Rexidamu et al., 2019[59]).

TMAO is linked to impaired renal function. In patients with compromised renal function absorbed from the intestine, TMA and TMAO may accumulate. Elevated levels of TMAO in patients with an impaired renal function may result from higher production or reduced clearance. Elevated levels of TMAO may be associated with type 2 diabetes (T2D) or atherosclerosis mediated renal dysfunction. This reduces the excretion of TMAO, thus increasing the levels of plasma TMAO (Zeisel and Warrier, 2017[89]). It means that TMAO may be a marker of disease, but not the direct causative factor of disease. Due to the increase in the number of TMAO-producing bacteria in plasma diabetic chronic kidney disease (CKD) patients, there was an increase in TMAO concentration compared to the control group (1.516 µg/mL vs. 0.183 µg/mL) (Al-Obaide et al., 2017[1]). In a similar study, CKD patients had higher median concentration of TMAO compared to controls (7.9 µM vs. 3.4 µM) (Hai et al., 2015[25]). Serum concentration of TMAO and TMA in subjects with reduced renal function, such as hemodialysis subjects indicates the importance of renal clearance for TMAO/TMA level regulation. In patients with more severely impaired kidney function, the levels of TMAO were significantly higher compared to the control group (77 µM vs. 2 µM), but not all of the study group. Only a few participants with severe impaired kidney function had significantly higher levels of TMAO. A change in the levels of TMAO concentration may cause individual variability or severity of the disease (Hai et al., 2015[25]). 

Researchers observed that levels of TMAO increased along with the body mass index, visceral adiposity index and fatty liver index in metabolic syndrome. The levels of TMAO concentration in the serum with values ≥ 8. 74 µM may be considered predictive of metabolic syndrome (Barrea et al., 2018[5]).

A correlation between increased TMAO levels and neurological disorders has been also hypothesized, but the role of TMAO in the central nervous system (CNS) has not been fully explored. Due to the importance of TMAO as a mediator of inflammatory processes, the possible participation of this compound in the etiology of neurological disorders is presumed. In the literature, there are very few studies demonstrating the relationship between brain disease and TMAO levels. The research conducted by Liu and Huang (2015[39]) suggested that in post-stroke patients to the progression of cerebral small vessel dysfunction elevated levels of TMAO may contribute due to the disruption of the blood-brain barrier by reducing the expression of claudin-5 and zonula occludens (ZO)-1 (Liu and Huang, 2015[39]). Villalobos and Renfro (2007[77]) suggested that TMAO suppresses stress-induced alteration of organic anion transport in the choroid plexus (Villalobos and Renfro, 2007[77]). Xu and Wang (2016[85]) demonstrated a new approach based on a model of genetic interaction to assess the brain-gut microbiome connections in Alzheimer's disease (AD). Moreover, they found common genetic pathways underlying AD biomarkers and TMAO. This approach showed a strong correlation between TMAO and Alzheimer's disease (Xu and Wang, 2016[85]).

## Application of Analytical Techniques in TMAO Determination in Biological Fluids

Various methods have been established to demonstrate TMAO and its related metabolites in body fluids (urine, plasma, serum, CSF, fecal sample). The analytical methods often used are chromatographic techniques, including liquid chromatography-mass spectrometry (LC-MS), high performance liquid chromatography-tandem mass spectrometry (HPLC-MS/MS), ultra-performance liquid chromatography-mass spectrometry (UPLC-MS), liquid chromatography/differential ion mobility spectrometry tandem mass spectrometry (HPLC/DMS-MS/MS), stable-isotope dilution-hydrophilic interaction liquid chromatography-time of flight mass spectrometry with multiple reaction monitoring (LC-ToF-MRM), gas chromatography-mass spectrometry (GC-MS), and also spectroscopic techniques such as proton nuclear magnetic resonance spectroscopy (^1^H-NMR). Table 1(Tab. 1) (References in Table 1: Awwad et al., 2016[3]; Bain et al., 2006[4]; Barrea et al., 2018[5]; Cho et al., 2017[11]; Enko et al., 2020[17]; Fiori et al., 2018[20]; Garcia et al., 2017[21]; Gessner et al., 2020[22]; Grinberga et al., 2015[24]; Heaney et al., 2016[27]; Jia et al., 2020[30]; Kadar et al., 2016[31]; Le et al., 2019[33]; Lee et al., 2010[34]; Li et al., 2017[37]; Missailidis et al., 2016[47]; Mueller et al., 2015[50]; Obeid et al., 2016[54]; Ocque et al., 2015[55]; Podadera et al., 2005[57]; Randrianarisoa et al., 2016[58]; Steuer et al., 2016[65]; Stubbs et al., 2016[66]; Wang et al., 2014[81]; Yang et al., 2021[86]; Yu et al., 2018[88]; Zheng et al., 2019[93]) summarizes the methods used to determine human TMAO in different body fluids using modern and advanced analytical techniques. Table 2(Tab. 2) (References in Table 2: Liu et al., 2016[38]; Mi et al., 2017[43]; Wang et al., 2020[78]; Wu et al., 2019[84]) presents the analytical techniques used to determine the levels of TMAO in mice.

## Conclusion

In conclusion, experimental findings constantly suggest that there is a potential link between the metabolites produced by the gut flora and the risk factors for diseases. Production of TMA and TMAO relies on intestinal microbiota, and also on host genetics, co-metabolism, and diet. To broaden our knowledge on nutrient metabolism and the ways diet may influence health, the research on the relationship between TMAO and intestinal microflora should be continued. If it was possible to fully exploit the potential of TMAO, it could become a new therapeutic target for improving the outcomes of patients. A better understanding of the specific role of bacteria in regulating the levels of TMAO concentration and their mechanism and reaction to dietary modulation, in combination with the factors determining the TMAO concentration is essential and necessary before the potential benefits of TMAO manipulation may be realized under the selected disease conditions.

## Conflict of interest

The authors declare no conflict of interest.

## Figures and Tables

**Table 1:**
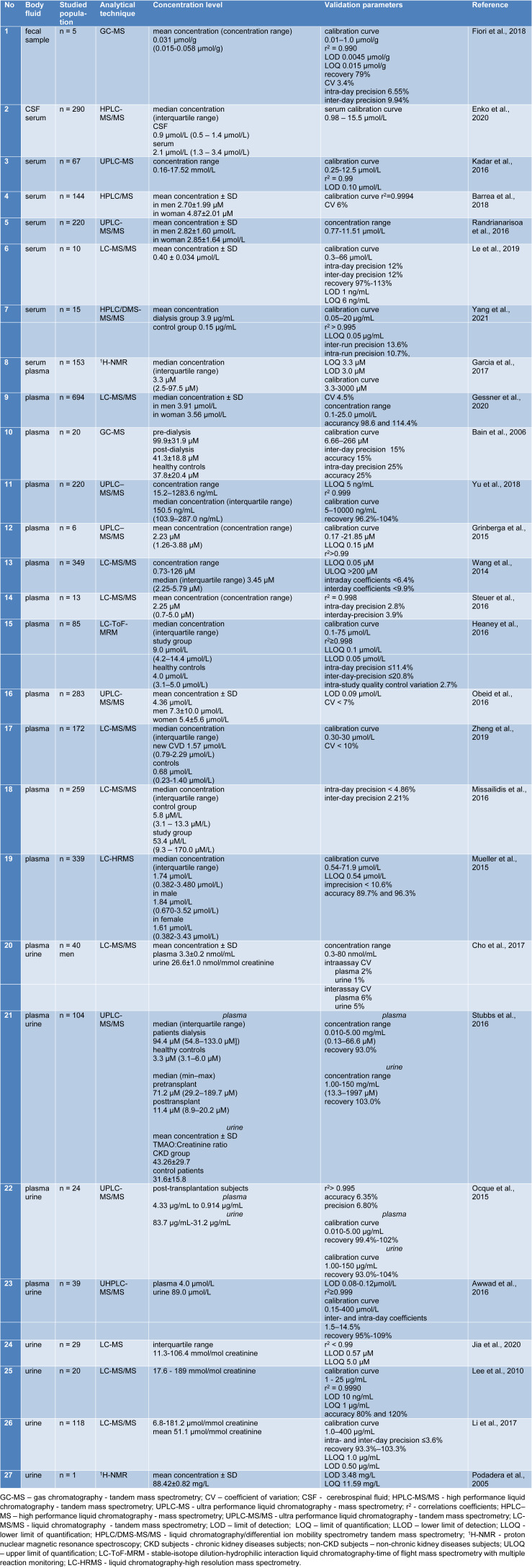
Methods of human TMAO determination in various body fluids using analytical techniques

**Table 2:**
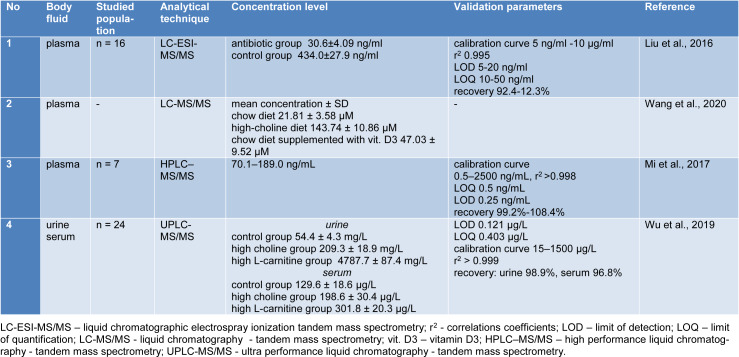
Methods of TMAO determination in mice in various body fluids using analytical techniques
